# Effect of electrostatic confinement on the dome-shaped superconducting phase diagram at the LaAlO_3_/SrTiO_3_ interface

**DOI:** 10.1038/s41598-024-77460-0

**Published:** 2024-10-30

**Authors:** Paweł Wójcik, Bartłomiej Szafran, Julian Czarnecki, Roberta Citro, Michał Zegrodnik

**Affiliations:** 1 Faculty of Physics and Applied Computer Science, AGH University of Krakow, Al. Mickiewicza 30, 30-059 Krakow, Poland; 2 Academic Centre for Materials and Nanotechnology, AGH University of Krakow, Al. Mickiewicza 30, 30-059 Krakow, Poland; 3 Department of Physics E. R. Caianiello, University of Salerno and CNR-SPIN, Via Giovanni Paolo II 132, Fisciano, SA Italy

**Keywords:** Oxide interfaces, Unconventional superconductivity, LAO/STO interface, Superconducting properties and materials, Surfaces, interfaces and thin films

## Abstract

The two-dimensional electron gas (2DEG) at the LaAlO$_3$/SrTiO$_3$ (LAO/STO) interface exhibits gate-tunable superconductivity with a dome-like shape of critical temperature as a function of electron concentration. This behavior has not been unambiguously explained yet. Here, we develop a microscopic model based on the Schrödinger–Poisson approach to determine the electronic structure of the LAO/STO 2DEG, which we then apply to study the principal characteristics of the superconducting phase within the real-space pairing mean-field approach. For the electron concentrations reported in the experiment, we successfully reproduce the dome-like shape of the superconducting gap. According to our analysis such behavior results from the interplay between the Fermi surface topology and the gap symmetry, with the dominant extended s-wave contribution. Similarly as in the experimental report, we observe a bifurcation effect in the superconducting gap dependence on the electron density when the 2DEG is electrostatically doped either with the top gate or the bottom gate. Our findings explains the dome-shaped phase diagram of the considered heterostucture with good agreement with the experimental data which, in turn, strongly suggest the appearance of the extended s-wave symmetry of the gap in 2DEG at the LAO/STO interface.

## Introduction

Since the discovery of superconductivity in two-dimensional electron gas (2DEG) at the interface between LaAlO$_3$ (LAO) and SrTiO$_3$ (STO)^1–6^ transition metal oxide heterostructures have gathered increasing interest as a natural platform for studying the interplay of superconductivity with magnetism^7–11^, spin-orbit interaction^12–18^, ferroelectricity^19–22^, and topological phenomena^23^. At the LAO/STO interface, all these phenomena can be well controlled by the field effect, leading to a complex phase diagram characterized by a dome-like shape of the superconducting state as a function of doping that resembles that observed in high $T_c$ cuprates, Fe-based superconductors, or heavy fermion systems^24–26^. For all class of these materials, the emergence of a critical point, above which the critical temperature ($T_c$) begins to decrease, is a hallmark of unconventional superconductivity and the corresponding atypical symmetry of the order parameters (d-wave in cuprates or $s^{\pm }$ for Fe-based materials, the most established ones). Note, however, that despite several theoretical proposals that have been reported to explain the characteristic dome-like shape of $T_c$ at the LAO/STO interface^3,5,27,28^, there is still no consensus on the physical origin of this phenomenon. One of the well-established model explains this behavior as resulting from the fragile $s_{\pm }$ superconducting state and the scattering processes between bands corresponding to opposite signs of the gap that lead to Cooper pair-breaking and suppression of the critical temperature^29,30^. This scenario relates the critical electron concentration ($n_{\textrm{c}}$), at which the maximum $T_c$ occurs, to the Lifshitz transition (LT) that separates the underdoped from the overdoped regime.

At the initial stage of research, superconductivity at the LAO/STO interface was believed to be predominantly associated with the low-lying $d_{xy}$ bands, while the observed decrease in $T_c$ was explained as occurring due to the population of the higher-lying $d_{xz/yz}$ bands^2,30^. Recent experiments, however, clearly indicate that the $d_{xy}$ bands play a negligible role, and superconductivity at the LAO/STO interface is induced rather by the population of the degenerate $d_{xz/yz}$ states with a high density of states which favours the emergence of the superconducting state.^3–5,14^. Those observations are in line with our recent model^31,32^, in which we have proposed that the characteristic dome of $T_c$ at the LAO/STO heterostructure arises from the interplay between a particular symmetry of the gap and the topology of the Fermi surface. Specifically, we have demonstrated that the characteristic shape of $T_c$ results from the induced extended s-wave symmetry of the order parameter, with the nodal lines gradually approached by the Fermi surface as the electron density increases. Interestingly, as reported in Ref.^33^, this specific symmetry is the one that favours the emergence of superconductivity at such low electron concentrations characteristic for transition metal oxide interfaces. This strongly suggests that the extended s-wave symmetry of the gap in the (001) LAO/STO 2DEG should be considered as a one of possible scenarios to explain the characteristic shape of $T_c$ with doping. For this purpose additional experimental efforts should be made to determine the gap symmetry and finally distinguish between both the aforementioned concepts.

The first step in this direction appears to have been taken by the latest experiment^34^ in which the authors used a double-gate field effect LAO/STO device, where electron doping can be separately controlled by the back and top gate. In this paper a bifurcation in the dependence of $T_c$ on the electron density, when applying either the top or the back gate, has been demonstrated. Although full calculations of the superconducting state are not included in Ref.^34^, the observed behaviour has been analyzed based on the electronic structure evaluated within the Schrödinger–Poisson approach, which demonstrates that the critical density above which $T_c$ decreases coincides with the population of the third excited $d_{xy}$ state (and the corresponding LT).

While these findings could be considered as supportive for the model based on intersubband scattering^29^, in this paper we demonstrate that all the observed features can be reproduced within the model based on the extended s-wave symmetry of the gap, resulting in the semi-quantitative agreement with the experimental data. For this purpose, we develop a full microscopic model in which the electronic structure of the system is calculated within the Schrödinger–Poisson approach, which is then employed in the calculations of the superconducting state within the real-space pairing model. For the electron concentration regime reported in the experiment^34^, we successfully reproduce the dome-like shape of $T_c$ and observe a bifurcation in the dependence of the energy gap on the electron density when the 2DEG is electrostatically doped either with the top gate or the back gate.

## Theoretical model

We consider 2DEG at the (100) LAO/STO interface in the double-gate device, similar to that reported in Ref.^34^. Although routinely, the back-gate architecture is used to control the 2DEG properties in STO-based interfaces^2,4,6,14^, the top-gating has been explored intensively in recent years^18,35,36^ as it is preferable to fabricate electrostatically defined nanostructures, i.e. quantum dots^37,38^ or quantum point contacts^39–41^. Importantly, in the LAO/STO 2DEG, the ability to control the spin-orbit coupling or $T_c$ by the top gate has been demonstrated to be comparable to that achieved for the back gating. It is well-established, that the electronic structure of 2DEG is governed by the 3*d* orbitals of Ti ions^42–44^. In our model we adopt the real space representation of the tight-binding Hamiltonian^12^ spanned by 3*d* Ti orbitals arranged in a square lattice, with the electronic structure of the interface determined based on the Schrödinger–Poisson approach within the effective mass approximation. Due to the computational demands related with numerical procedure of determining the superconducting gaps in multiband systems, our model assumes that the bands are independent, i.e. we neglect the spin-orbit coupling and hybridization between the $d_{xz}$ and $d_{yz}$ bands. As shown in our recent paper^32^, in the considered real-space pairing model, both those effects have negligible impact on the gap symmetry and do not significantly alter the dependence of $T_c$ on the electron density.

### Schrödinger–Poisson approach

The electronic structure of the LAO/STO 2DEG under top/back gating is calculated using a numerical method that considers crucial factors, such as the anisotropy of the 3*d* bands and the electrostatic environment associated with the applied gate. For this purpose, we utilize a standard Schrödinger–Poisson approach, assuming translational invariance in the *xy*-plane, i.e., $\psi _{l,n}(\textbf{r})=\phi _{l,n}(z)e^{i(k_xx+k_yy)}$, where $l=xy, yz, xz$ represents the 3*d*-type orbitals. The Schrödinger–Poisson model tailored to the LAO/STO interface was described in detail in Refs.^45–47^, and was successfully used in the past to analyze confinement at the interface, explain phase separation, and propose new trapping mechanisms at the LAO/STO interface. According to this model, the out-of-plane component $\phi _{l,n}(z)$ can be determined from the Schrödinger equation which, in the effective mass approximation, is given by

1 $$\begin{aligned} \left[ -\frac{\hbar ^2}{2m_l^z}\frac{d^2}{dz^2} + V(z) \right] \phi _{l,n}(z;k_x,k_y)=\left( E_{l,n}+\frac{\hbar ^2 k_x^2}{2m_l^x} +\frac{\hbar ^2 k_y^2}{2m_l^y} \right) \phi _{l,n}(z;k_x,k_y), \end{aligned}$$

where $m_l^{x(y,z)}$ is the effective mass of electrons in the orbital *l*, in the *x*(*y*, *z*) direction. Due to the symmetry of 3*d* orbitals we assume $m_{xy}^{x(y)}=m_{xz}^{x(z)}=m_{yz}^{y(z)}=m_l, \,\, m_{xy}^{z}=m_{xz}^{y}=m_{yz}^{x}=m_h$, where $m_{l(h)}$ corresponds to the mass of light and heavy electrons that leads to the anisotropy of the $d_{xz/yz}$ orbitals in the $k_x-k_y$ plane.

Since the conduction band offset between LAO and STO is approximately 1 eV (both are insulators with band gaps of 5.6 eV and 3.2 eV, respectively ^48^), in the low-energy limit, we can treat the interface as an infinite energy barrier, taking the boundary condition for Eq. (1) in the form $\phi (0)=0$ and $\phi (L)=0$, where *L* represents the length of the system used in the calculations.

The self-consistent potential *V*(*z*) in Eq. (1) is evaluated at the mean field level by solving of the Poisson equation

2 $$\begin{aligned} \frac{d}{dz} \left( \varepsilon _r(z)\frac{d}{dz} \right) V(z)= -\frac{n_e(z)+n_t(z)}{\varepsilon _0}, \end{aligned}$$

where $n_t(z)$ is the density of trapped electrons which emerges in the system to maintain charge neutrality in response to positive countercharges^16,45,46^ arising from oxygen vacancies or polarity catastrophe^42–44^. The spatial distribution of $n_t(z)$ is the input of the Schrödinger–Poisson model and it is taken in the form^34^

3 $$\begin{aligned} n_t(z)=n_t^0\exp \left( - \frac{z}{L_t} \right) \end{aligned}$$

where $n_t^0$ determines the density of trapped charge at $z=0$, and $L_t$ is a characteristic length of the trapped charge distribution, typically ranging between 10 and 100 nm^16,45,46^. Note that the inclusion of trapped charge is crucial for obtaining a confinement at the interface as its absence may lead to an unstable solution with the Fermi energy being located above the top of the quantum well.

Since the dielectric constant of STO strongly depends on temperature and electric field, in Eq. (2) we utilize a spatially dependent $\varepsilon (z)$, which varies according to the following formula, applicable for low temperatures^34^

4 $$\begin{aligned} \varepsilon _r=\varepsilon _{r}(0)+\frac{1}{A+B|F|}, \end{aligned}$$

where *F* is the electric field, $A=4.097\times 10^{-5}$, $B=4.907\times 10^{-10}$ m/V and $\varepsilon _{r}(0)=100$.

Finally, the electron density $n_e(z)$ in the quantum well is given by

5 $$\begin{aligned} n_e(z)=\sum _{l,n} |\phi _{l,n}(z)|^2 f_{2D}(E_{l,n},\mu ;k_x,k_y)=\sum _{l,n} |\phi _{l,n}(z)|^2 \frac{\sqrt{m_l^xm_l^y}k_BT}{2 \pi } \ln \left( 1+\exp \left( \frac{E_{l,n}-\mu }{k_BT} \right) \right) , \end{aligned}$$

where $f_{2D}(E_{l,n},\mu ;k_x,k_y)$ represents the Fermi-Dirac distribution integrated over the wave vector component $k_x (k_y)$ corresponding to the translational symmetry.

Equation (2) is solved under boundary conditions that depend on the top/back gating and are described in details in Ref.^45^. Specifically, $V(L)=0$ or $V(L)=\alpha V_g$ for the voltage $V_g$ applied to the top or back gate, respectively. Here, $\alpha$ represents the lever arm that transforms the voltage applied to the back gate into the potential induced at the boundary of the numerical box, *L*. In the simplest form, assuming the ohmic approximation, $\alpha =L/L_{tot}$, where $L_{tot}$ is the total length of the STO substrate.

The boundary condition at the LAO/STO interface, at $z=0$, can be formulated based on the charge neutrality which leads to the condition

6 $$\begin{aligned} \varepsilon _r(0) \frac{dV}{dz} |_{z=0}=\frac{en_{tot}}{\varepsilon _0}, \end{aligned}$$

where the total charge density, $n_{tot}=\int (n_e(z)+n_t(z))dz$.

In the Schrödinger–Poisson approach Eqs. (1) and (2) are solved alternatively until the self-consistency is reached, which we consider to occur when the relative variation of the potential between two consecutive iterations is lower than $10^{-5}$ eV. In each iteration, a potential profile *V*(*z*), electric permittivity $\varepsilon _r(z)$, electron density $n_e(z)$ as well as electronic structure $E_{l,n}$ are determined whereas the latter is then utilized to evaluate the superconducting properties of the 2DEG. Calculations have been conducted for a specified total electron density corresponding to gate voltages (extracted from the experiment^34^), while the chemical potential is determined to achieve the desired $n_{e}^{2D}=\int n_e(z)dz$.

If not stated otherwise, calculations have been carried out using the following parameters: $n_t^0=5\times 10^{13}$ $\hbox {cm}^{-2}$, $L_t=15$ nm, $\alpha =0.005$, and $L=100$ nm. The model we utilized is similar to that applied in Ref.^34^, with one important difference that the masses $m_l$ and $m_h$ are treated as parameters which may vary in appropriate ranges chosen based on experimental reports - for more details, see the discussion in Results.

### Superconducting gap calculations

Superconducting properties of LAO/STO 2DEG are analyzed within the mean field approximation justified due to the low electron concentration typical for transition metal oxide heterostructures. For this purpose, we consider the real space pairing scenario defined on the tight-binding Hamiltonian^12^ spanned by 3*d* Ti orbitals arranged in a square lattice. The Hamiltonian of the system is given by

7 $$\begin{aligned} \hat{H}_=\sum _{\textbf{k}ln\sigma } \varepsilon _{l,n}(\textbf{k}) \hat{c}^{\dagger }_{\textbf{k},l,n,\sigma } \hat{c}_{\textbf{k},l,n,\sigma }+\hat{H}_{SC}, \end{aligned}$$

where $\hat{c}^{\dagger }_{\textbf{k},l,n,\sigma }(\hat{c}_{\textbf{k},l,n,\sigma })$ creates (annihilates) electrons with spin $\sigma$, momentum $\textbf{k}$ in the n-the state of the orbital $l=xy,xz,yz$.

In Eq. (7), $\varepsilon _{l,n}(\textbf{k})$ defines the dispersion relation which for a given orbital *l* can be expressed as

8 $$\begin{aligned} \varepsilon _{{xy,n}} ({\mathbf{k}}) & = E_{{xy,n}} + 4t_{l} - 2t_{l} \cos k_{x} - 2t_{l} \cos k_{y} , \\ \varepsilon _{{xz,n}} ({\mathbf{k}}) & = E_{{xy,n}} + 2t_{l} + 2t_{h} - 2t_{l} \cos k_{x} - 2t_{h} \cos k_{y} , \\ \varepsilon _{{yz,n}} ({\mathbf{k}}) & = E_{{xy,n}} + 2t_{l} + 2t_{h} - 2t_{h} \cos k_{x} - 2t_{l} \cos k_{y} , \\ \end{aligned}$$

where $E_{l,n}$ are energies of the subsequent electronic states for the orbital *l* calculated within the Schrödinger–Poisson approach. We determine the energy of $N=50$ lowest states for each orbital, with the maximum energy significantly exceeding the chemical potential. The correspondence between the effective masses used in the Schrödinger–Poisson model to the parameters $t_l(t_h)$ of tight-binding Hamiltonian (8) is given by $m_{l(h)}=\hbar ^2/2a^2t_{l(h)}$, where *a* is the lattice constant, $a=0.39$ nm.

In our model, the superconducting state is induced by a real-space intersite intraorbital pairing between the nearest neighboring sites as well as the interorbital pair hopping term. $\hat{H}_{SC}$ in Eq. (7) has the form

9 $$\begin{aligned} \hat{H}_{SC}=-J\sum _{\langle ij \rangle ln\sigma }\hat{c}^{\dagger }_{i,l,n,\sigma }\hat{c}^{\dagger }_{j,l,n,\overline{\sigma }}\hat{c}_{i,l,n,\overline{\sigma }}\hat{c}_{j,l,n,\sigma } -J^{\prime }\mathop {{\mathop {\sum }\nolimits ^{\prime }}}\limits _{\langle ij \rangle ll^{\prime }nn^{\prime }\sigma}\hat{c}^{\dagger }_{i,l,n,\sigma }\hat{c}^{\dagger }_{j,l,n,\overline{\sigma }}\hat{c}_{i,l^{\prime },n^{\prime },\overline{\sigma }}\hat{c}_{j,l^{\prime },n^{\prime },\sigma }, \end{aligned}$$

where the summations run over the nearest-neighbors only, $\overline{\sigma }$ is the spin opposite to $\sigma$, and the pairing strength is determined by the parameters *J* and $J^{\prime }$. In the calculations, we assume that the interorbital pair hopping energy, $J^{\prime }$, is one order of magnitude smaller than the intraorbital coupling constant, *J*.

Since in our model we neglect the coupling between orbitals (which may be induced by the SOC or hybridization) we can describe our 3*N* multiband system by a sum of 3*N* Hamilonians $2\times 2$ defined in the Nambu space. Within the standard mean field approach, Hamiltonian (7) can be transformed to

10 $$\begin{aligned} \begin{aligned} \hat{H}=&\sum _{\textbf{k}ln}{\hat{\textbf{f}}}^{\dagger }_{\textbf{k},l,n}{\hat{\textbf{H}}}_{\textbf{k},l,n}{\hat{\textbf{f}}}_{\textbf{k},l,n}+ \sum _{\textbf{k}ln} (\varepsilon _{\textbf{k},l,n}-\mu ) +\sum _{\langle ij \rangle l n \sigma } J \langle \hat{c}_{i,l,n,\sigma }\hat{c}_{j,l,n,\overline{\sigma }}\rangle ^2 + \sum _{\langle ij \rangle ll^{\prime } n n^{\prime }\sigma } J^{\prime }\langle \hat{c}_{i,l,n,\sigma }\hat{c}_{j,l,n,\overline{\sigma }}\rangle \langle \hat{c}_{i,l^{\prime },n^{\prime },\sigma }\hat{c}_{j,l^{\prime },n^{\prime }, \overline{\sigma }^{\prime }} \rangle , \end{aligned} \end{aligned}$$

where the vector ${\hat{\textbf{f}}}_{\textbf{k},l,n}=(\hat{c}^{\dagger }_{\textbf{k},l,n,\uparrow },\hat{c}^{\dagger }_{-\textbf{k},l,n,\downarrow })^T$ and

11 $$\begin{aligned} {\hat{\textbf{H}}}_{\textbf{k},l,n} = \left( \begin{array}{cc} \varepsilon _{l,n}(\textbf{k})-\mu & \Gamma _{l,n} \\ \Gamma _{l,n}^\dagger & -(\varepsilon _{l,n}(-\textbf{k})-\mu ) \end{array} \right) . \end{aligned}$$

In the above, the pairing energy

12 $$\begin{aligned} \Gamma _{l,n}= \sum _{\langle j(i)\rangle } \Delta _{i,j,l,n}^{\sigma ,\overline{\sigma }} e^{i\textbf{k}\cdot \textbf{R}_{ij}}, \end{aligned}$$

where $\textbf{R}_{ij}=\textbf{R}_{i}-\textbf{R}_{j}$ and the summation runs over the four nearest-neighboring atomic sites surrounding the *i*-th atomic position. Notice that the value of $\Gamma _{ln}$ is spatially independent due to homogeneity of the system. The explicit definition of the pairing amplitudes $\Delta _{i,j,l,n}^{\sigma \overline{\sigma }}$ is given by

13 $$\begin{array}{*{20}c} {\Delta _{{i,j,l,n}}^{{\sigma \bar{\sigma }}} = - J\langle \hat{c}_{{i,l,n,\sigma }} \hat{c}_{{j,l,n,\bar{\sigma }}} \rangle - J^{\prime } \sum _{{\begin{array}{*{20}c} {l^{\prime } (l^{\prime } \ne l)} \\ {n^{\prime } (n^{\prime } \ne n)} \\ \end{array} }} \langle \hat{c}_{{i,l^{\prime } ,n^{\prime } ,\sigma }} \hat{c}_{{j,l^{\prime } ,n^{\prime } ,\bar{\sigma }}} \rangle .} \\ \end{array}$$

In our approach we do not impose any constraints on the pairing amplitudes. Instead, the resulting real-space amplitudes, that are the result of the self-consistent calculations, are decomposed into the symmetry resolved counterparts representing the three basic symmetries: s, p, and d. These symmetries are determined from Eq. (12) by using the values of $\Delta _{i,j,l,n}^{\sigma ,\overline{\sigma }}$ and they take the form

14 $$\begin{aligned} \Delta ^{\sigma \bar{\sigma }|s,p,d}_{l,n}=\frac{1}{4}\sum _{\langle j(i) \rangle }\gamma ^{s,p,d}_{ij}\Delta _{i,j,l,n}^{\sigma \overline{\sigma }},\quad \end{aligned}$$

with the real-space symmetry factors

15 $$\begin{aligned} \gamma _{{ij}}^{s} & = (\delta _{{{\mathbf{R}}_{{ij}} - \hat{x}}} + \delta _{{{\mathbf{R}}_{{ij}} + \hat{x}}} + \delta _{{{\mathbf{R}}_{{ij}} - \hat{y}}} + \delta _{{{\mathbf{R}}_{{ij}} + \hat{y}}} ), \\ \gamma _{{ij}}^{d} & = (\delta _{{{\mathbf{R}}_{{ij}} - \hat{x}}} + \delta _{{{\mathbf{R}}_{{ij}} + \hat{x}}} - \delta _{{{\mathbf{R}}_{{ij}} - \hat{y}}} - \delta _{{{\mathbf{R}}_{{ij}} + \hat{y}}} ), \\ \gamma _{{ij}}^{{p_{x} }} & = (\delta _{{{\mathbf{R}}_{{ij}} - \hat{x}}} - \delta _{{{\mathbf{R}}_{{ij}} + \hat{x}}} ), \\ \gamma _{{ij}}^{{p_{y} }} & = (\delta _{{{\mathbf{R}}_{{ij}} - \hat{y}}} - \delta _{{{\mathbf{R}}_{{ij}} + \hat{y}}} ). \\ \end{aligned}$$

In the above, $\delta _{\textbf{v}}$ is the appropriate Kronecker delta, giving $\delta _{\textbf{v}}=1$ only when $\textbf{v}=(0,0)$ and $\hat{x} (\hat{y})$ is the unit vector along *x*(*y*) axis. It is important to note that the *s*- and *d*-wave gap symmetries are compatible with the spin-singlet pairing ($\Delta ^{\uparrow \downarrow }=-\Delta ^{\downarrow \uparrow }$), while spin-triplet pairing ($\Delta ^{\uparrow \downarrow }=\Delta ^{\downarrow \uparrow }$) is allowed for the *p*-wave gap.

In our calculations the pairing amplitudes $\Delta _{i,j,l,n}^{\sigma \overline{\sigma }}$ are determined in a self-consistent manner by solving the set of Eqs. (11) and (13), and in general a multicomponent gap structure can be realized with a mixture of *s*, *p*, and *d* symmetries as well as both the spin singlet and triplet. Their particular contributions results from the minimalization of the free energy under the condition that the superconducting state is thermodynamically stable - its free energy is lower than the energy of a normal state.

## Results

**Fig. 1:**
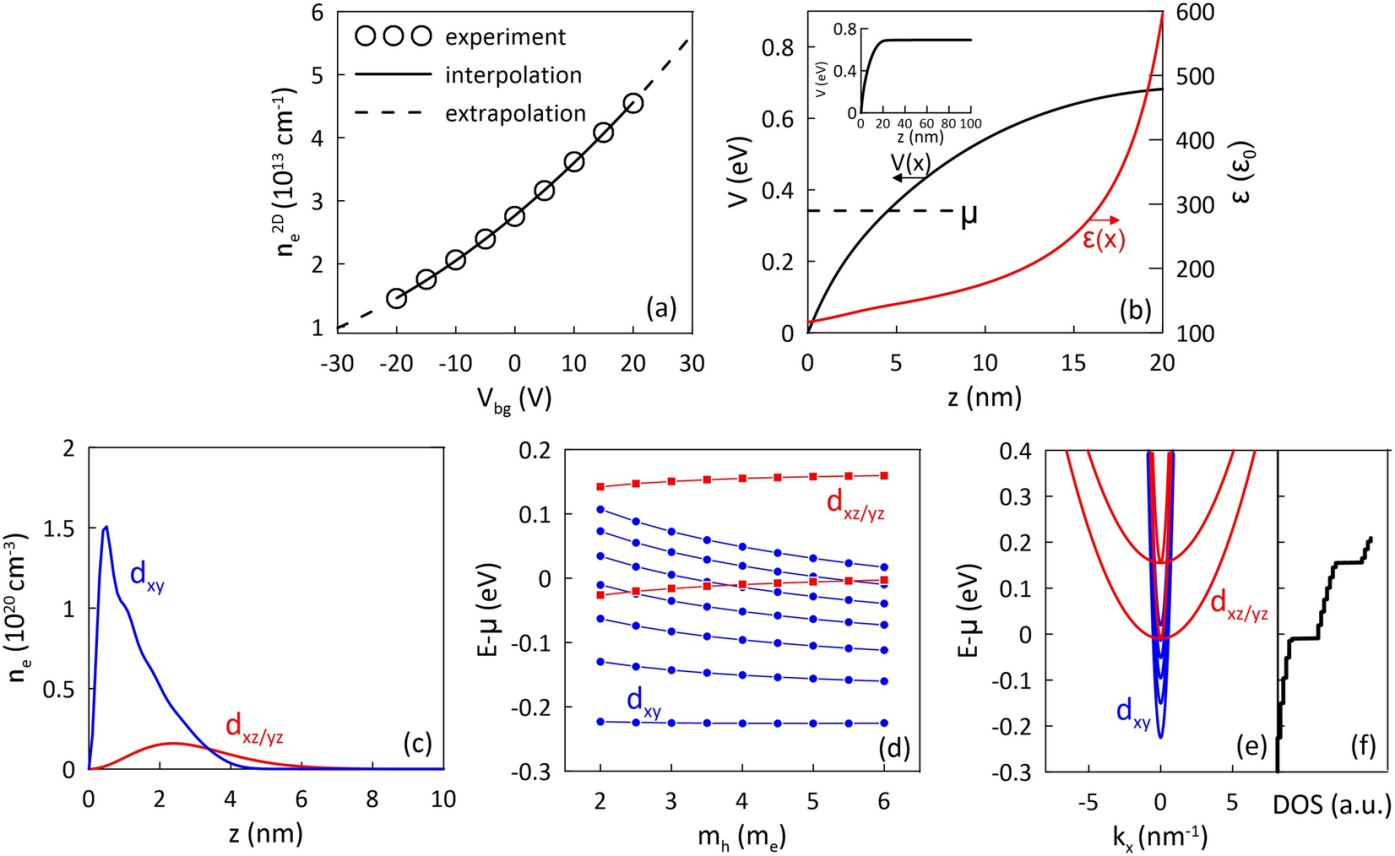
Results of the Schrödinger–Poisson calculations for $V_{bg}=0$ and $m_l=0.1m_e$. (**a**) 2D electron density $n_e^{2D}$ vs. back-gate voltage $V_{bg}$ extracted from the experimental data (Fig. 3 in Ref.^34^). Experimental points are marked by dots and the line represents the interpolation. The data are extrapolated by $\pm 10$ V relative to the voltage range of $[-20,20]$ V reported in the experiment - marked by dashed lines. (**b**) Self-consistent potential *V*(*z*) (black line) and relative permittivity $\varepsilon$ (red line) profiles in the close vicinity of the LAO/STO interface. In the inset, the potential profile *V*(*z*) is plotted over the entire simulated range with $L=100$ nm. (**c**) Electron density distribution near the LAO/STO interface, divided into $d_{xy}$ and $d_{xz/yz}$ bands. (**d**) Dependence of the electronic structure on the electron mass $m_h$. (**e**) Dispersion relation *E*(*k*) together with the density of states DOS (**f**). Results in panels (**b**, **c**, **e**, **f**) evaluated for $m_h=4.0m_e$.

Here, based on the microscopic model defined in the previous section, we demonstrate that the characteristic dome-like shape of $T_c$ in the LAO/STO 2DEG, and the observed bifurcation in the dependence of $T_c(n_e^{2D})$^34^ can be explained as resulting from the specific symmetry of the gap, preferable in the low electron density systems. For this purpose, we first discuss the impact of the back-gating on the electrostatic confinement and then we analyze its correspondence to superconducting properties of 2DEG. In the next step, we study the impact of the top gate voltage and demonstrate a bifurcation of the superconducting gap with respect to the electrostatic doping with a back- or a top-gate.

### Electrostatic confinement

To reflect the realistic electron densities measured in the experiment, in the calculations we adopt the dependence $n_{e}^{2D}(V_{bg})$ from Fig. 3 of Ref.^34^. In Fig. 1a, we display the 2D electron density $n_e^{2D}$ as a function of the back gate voltage $V_{bg}$, extracted from the experiment. The data are extrapolated by $\pm 10$ V relative to the voltage range of $[-20,20]$ V reported in the paper^34^—marked by dashed lines in the figure. Note that the critical parameters, that determine the electronic structure of the LAO/STO 2DEG, are the effective masses $m_{l(h)}$ corresponding to light and heavy electrons, which in turn translate into $t_{l(h)}$ in the real space model. The values of $m_{l(h)}$ at the LAO/STO interface are not well established and vary across different studies: $m_h=14m_e$ and $m_l=0.7m_e$ in Ref.^16^, the isotopic scenario with $m_l=m_h=3.14m_e$ is assumed in Ref.^34^ or $m_l=0.28m_e$ and $m_h=6.26m_e$ are given in Ref.^12^. Since now it is well established that the onset of superconductivity corresponds to the population of the $d_{xz/yz}$ bands^3–5,14^, the values of $m_{l(h)}$ in our calculations are determined to satisfy this condition. Based on the experimental phase diagram (Fig. 3 in Ref.^34^), which exhibits the onset of superconductivity at $n_e=1.45 \times 10^{13}$ $\hbox {cm}^{-3}$ for the back-gate voltage $V_{bg}\approx -20$ V, we set $m_l=0.1m_e$ and $0.05m_e$ and consider different values of $m_h$ varying from $2m_e$ to $6m_e$. Notably, the chosen values of the parameters are close to those reported by Diez et al.^12^, extracted from the DFT calculations.

**Fig. 2:**
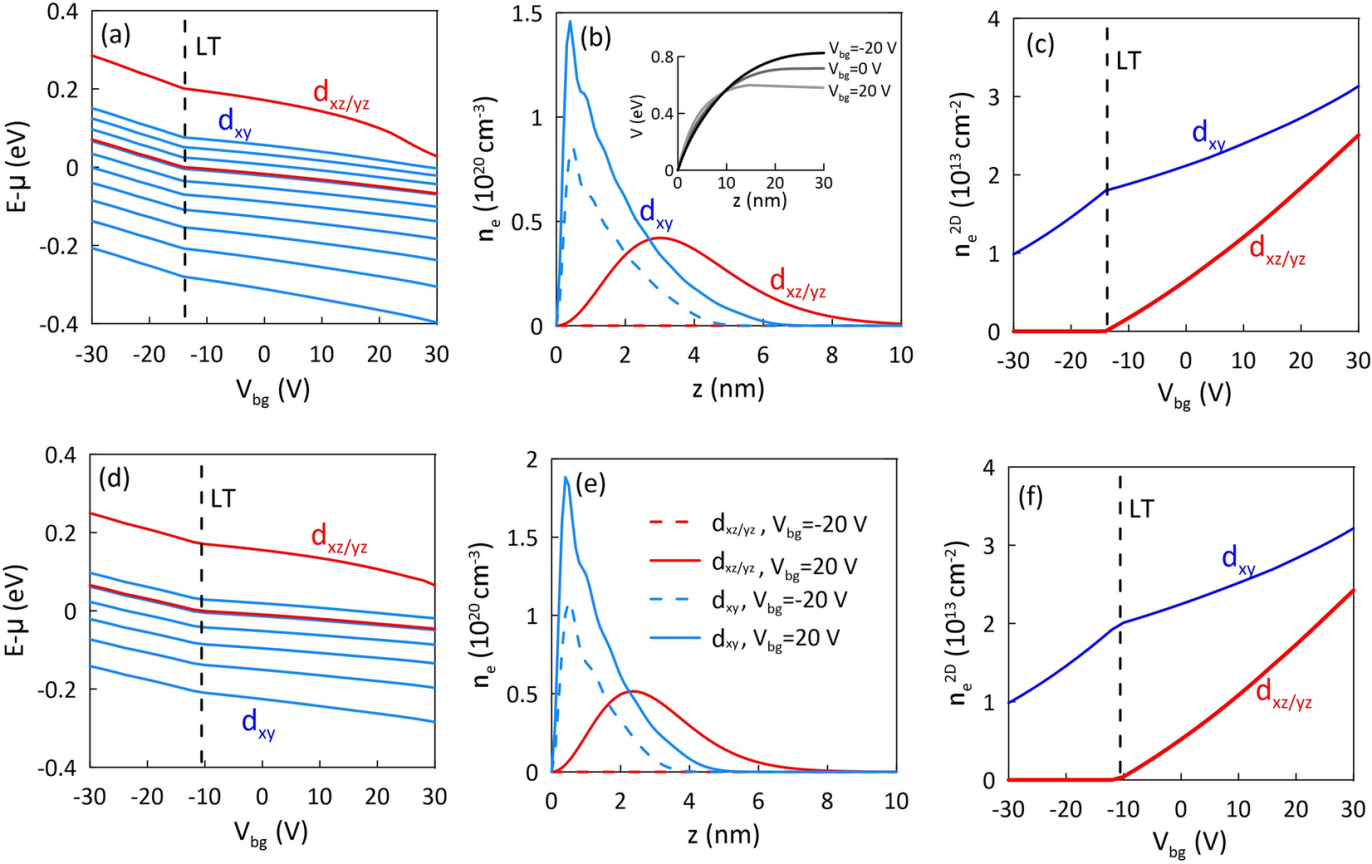
Results of the Schrödinger–Poisson calculations under the back-gating. (**a**, **d**) Electronic structure vs. back-gate voltage, $V_{bg}$. (**b**, **e**) Electron density profiles near the LAO/STO interface divided into $d_{xy}$ and $d_{xz/yz}$ orbitals, calculated for $V_{bg}=-20$ V and 20 V. In the inset of panel (**b**), the potential profile *V*(*z*) is plotted for $V_{bg}=-20,0,20$ V. (**c**, **f**) The electron density $n_e^{2D}$ for the $d_{xy}$ and $d_{xz/yz}$ orbitals on the back gate voltage $V_{bg}$. Results for $m_l=0.05m_e$ (**a**–**c**) and $m_l=0.1m_e$ (**d**–**f**).

Results of the Schödinger–Poisson calculations for $V_{bg}=0$ corresponding to $n^{2D}_e=2.75\times 10^{13}$ $\hbox {cm}^{-2}$ and $m_l=0.1m_e$ are shown in Fig. 1b–f. In panel (b), we present the self-consistent potential profile (black line) in the position range corresponding to the immediate vicinity of the interface (up to 20 nm). The full potential across the entire simulation range is presented in the inset of panel (b). We can see that, near the LAO/STO interface, the potential forms a narrow triangular quantum well with a typical dependence of eigenenergies being inversely proportional to the particle mass and to the confining dimension. The chemical potential $\mu$, in panel (b) marked by the dashed horizontal line, lies well below the top of the quantum well preventing the leakage of electrons into continuum of states in the STO substrate.

In Fig. 1c, we present the electron density distribution near the interface, for different types of orbitals. Importantly, the $d_{xy}$ and $d_{xz/yz}$ electrons forming 2DEG are confined within less then 10 nm at the interface, which is consistent with the reported spatial distribution of electrons at the LAO/STO heterostructure^49,50^. Their strong localization near the interface reduces the electric permittivity of the 2DEG. Although the dielectric constant of STO can reach values as high as 20,000 in the bulk, in Fig. 1b (red line) we can see that near the interface where the 2DEG is formed, its value is limited by the strong electric field [see Eq. 4], reaching values in the moderate range of 100–200 $\varepsilon _0$. Note, that due to the characteristic orientations of *d* orbitals and the corresponding heavy and light mass of electrons in the *z*-direction, the 2DEG is formed by four $d_{xy}$ states and only one $d_{xz/yz}$ state for the chosen $m_l=0.1m_e$ - see the $E(k_x)$ relation presented in Fig. 1e. The density of states (DOS) as a function of energy, calculated based on the determined electronic structure, is presented in Fig. 1f. The high effective mass of the $d_{xz/yz}$ bands in the $k_{x,(y)}$ direction, leads to an abrupt increase of DOS when the single $d_{xz/yz}$ state is populated. The relative contribution to DOS, being negligibly low for $d_{xy}$ states and dominant for $d_{xz/yz}$, is crucial to understand the appearance of superconducting phase discussed in the next subsection. Here, it is important to underline that the first $d_{xz/yz}$ excited state is located higher at the energy scale, significantly above the chemical potential, so even the applied voltage does not lead to the population of this state. From Fig. 1d, which presents the energies of states as a function of the mass of heavy electrons $m_h$, one can conclude that this characteristic electronic structure with a single $d_{xz/yz}$ state near the Fermi level is not affected by changes in $m_h$ over a wide regime.

Now, let us analyze the modification of the electronic structure when we apply voltage to the back gate. Figure 2 presents results of the Schrödinger–Poisson calculations performed for the light effective mass $m_l=0.05m_e$ (a–c) and $m_l=0.1m_e$ (d–f). Note that the results for the chosen $m_l$ do not exhibit any significant difference in behavior under the application of the back gate voltage, except that for $m_l=0.05m_e$ the number of $d_{xy}$ states forming the 2DEG at $V_{bg}=0$ increases up to seven—compare Fig. 2a,d. In this case, still only a single $d_{xz/yz}$ state is populated, leading to a high DOS at $V_{bg}=0$ (not shown here).

When applying a voltage $V_{bg}$, the electrostatic potential, after increasing in the region close to the interface, decreases (or increases) linearly with a distance for positive (or negative) back gate voltage, as shown in the inset of Fig. 2b. For $V_{bg}>0$, the electron concentration for a specific orbitals is slightly pushed away from the interface, as shown in Fig. 2b,e, which presents the electron density distributions for $d_{xy}$ and $d_{xz/yz}$ states at different back gate voltages. Note that for $V_{bg}>0$, the electrons with energy close to the top of the quantum well, including the single $d_{xz/yz}$ state, are weakly confined and may escape and localize on the right side of the simulation box. Thus, especially for positive back-gating, the quantum well near the LAO/STO interface remains stable only due to the trapped charge near the interface which in our case was determined in such a way to cover the entire experimentally accessible voltage regime without releasing of $d_{xz/yz}$ electrons from the 2DEG.

**Fig. 3:**
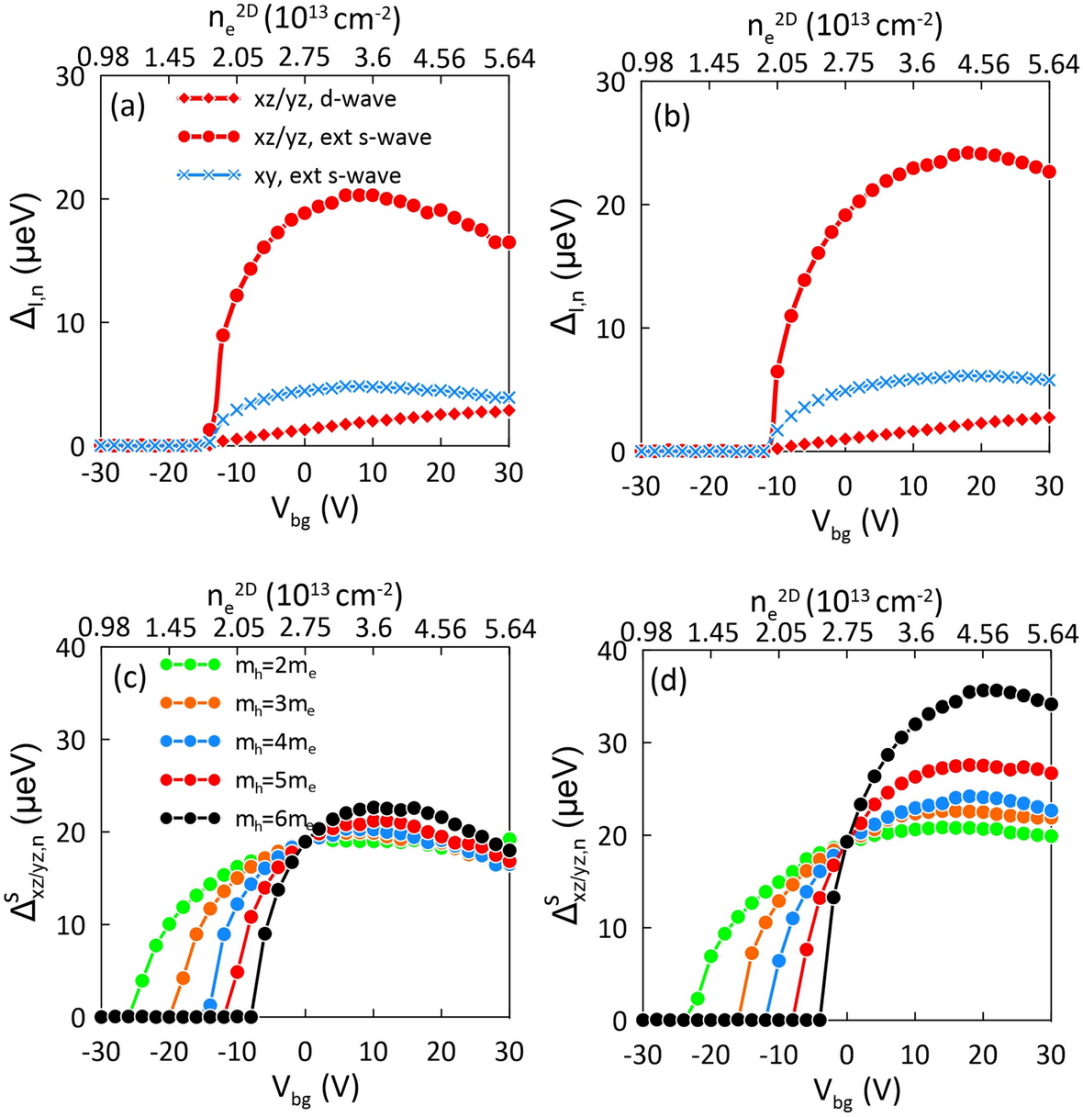
(**a**, **b**) Superconducting energy gap $\Delta _{l,n}$ as a function of back-gate voltage, $V_{bg}$. The upper *x* axis displays the corresponding 2D electron density extracted from the experimental data (cf. Fig. 3 in Ref.^34^). Panels (**c**, **d**) present the dominant extended s-wave component of superconducting gap in the $d_{xz/yz}$ bands for different strength of electronic band anisotropy determined by $m_h$. Results for (**a**, **c**) $m_l=0.05m_e$ and (**b**, **d**) $m_l=0.1m_e$.

Figure 2a,d show the energy of electronic states, determined with respect to the Fermi energy, as a function of the back gate voltage. We see that the change in the confinement potential related to back-gating results in decreasing in energy of states, with the $d_{xz/yz}$ crossing the Fermi level at the voltage range $[-20V,-10V]$ depending on $m_h$—Fig. 2a,d show results for $m_h=4m_e$. Due to the high DOS of $d_{xz/yz}$ orbitals, at the crossing point (LT), the electrostatic environment changes so significantly that the monotonicity of $E(V_{bg})$ are modified substantially. Similar behavior is observed in the dependence of the electron concentration in the 2DEG divided into orbitals as a function of $V_{bg}$, presented in Fig. 2c,f. Here, the population of $d_{xz/yz}$ leads to decrease of the slope in $n_e^{2D}(V_{bg})$ for the $d_{xy}$ orbitals, in agreement with the experimental observations ^2,6,40^.

### Superconducting properties

The electronic structure determined from the Schrödinger–Poisson approach has been then used to evaluate the superconducting energy gap and its evolution under the application of back-gate voltage. For this purpose, we have used the multiband model described in Theoretical Model, treating the pairing energy *J* as a parameter chosen to achieve the energy gap in the $d_{xz/yz}$ band at the level of 20 $\mu$eV at $V_{bg}=0$. This value is close to that reported in the experiment^51^ and our previous theoretical studies^31,32^.

**Fig. 4:**
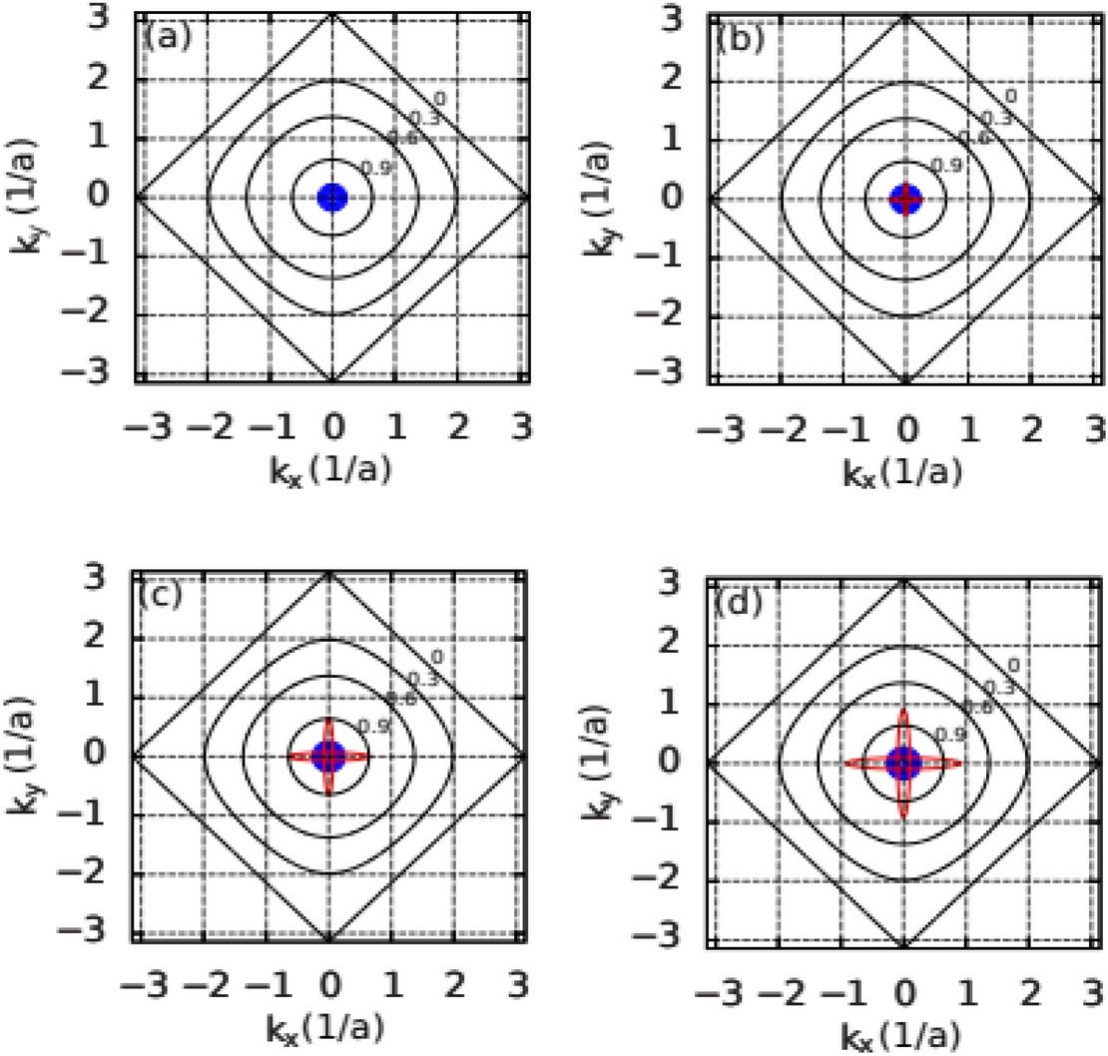
Fermi surfaces of the $d_{xz/yz}$ (red) $d_{xy}$ (blue) bands plotted on the map of isolines determined for the extended s-wave symmetry factor corresponding to $\gamma (\textbf{k}) = 0.9, 0.6, 0.3, 0.0$ (gray lines). Results are shown for (**a**) $V_{bg}=20$ V, where the superconducting phase is not observed, (**b**) $V_{bg}=-10$ V, just above the superconductivity onset, (**c**) $V_{bg}=10$ V, at the $\Gamma _{l,n}$ maximum and above the maximum, for (**d**) $V_{bg}=20$ V in the regime of decreasing $\Gamma _{l,n}$.

In Fig. 3a,b, we present the superconducting amplitudes $\Delta _{l,n}$ as a function of the back-gate voltage, calculated for (a) $m_l=0.05m_e$ and (b) $m_l=0.1m_e$ for $m_h=4m_e$. The corresponding 2D electron densities are displayed on the upper *x* axis and match those extracted from the experiment^34^. As shown in Fig. 3a,b, the extended s-wave pairing amplitude in the doubly degenerate $d_{xz/yz}$ state constitutes the dominant contribution to the superconducting phase and it reproduces the characteristic dome-like shape as a function of the electron concentration, reported in the experiment^34^. The small contribution of the *d*-wave symmetry is a results of a strong anisotropy of the $d_{xz/yz}$ bands in the $k_x-k_y$ plane. Note that for the isotropic $d_{xy}$ bands, the *d*-wave component does not occur. In this case, the pronounced extended *s*-wave amplitude is equal for each of the occupied states as it mainly results from the Cooper pair hopping from the upper $d_{xz/yz}$ bands. Importantly, due to low DOS, inducing superconductivity in the $d_{xy}$-bands is not possible independently of the $d_{xz/yz}$ bands. Consequently, in our model, the superconductivity is solely induced by the occupation of the $d_{xz/yz}$ bands, which is in line with recent experimental findings^3–5,14^. Finally, the evolution of the $\Delta _{l,n}(V_{bg})$ dependence with the $d_{xz/yz}$ band anisotropy, determined by $m_h$, is displayed in Fig. 3c,d for $m_l=0.05m_e$ and $0.1m_e$, respectively. These results indicate that the dome is more pronounced for larger $m_h$, corresponding to the strong anisotropy of the $d_{xz/yz}$ bands.

**Fig. 5:**
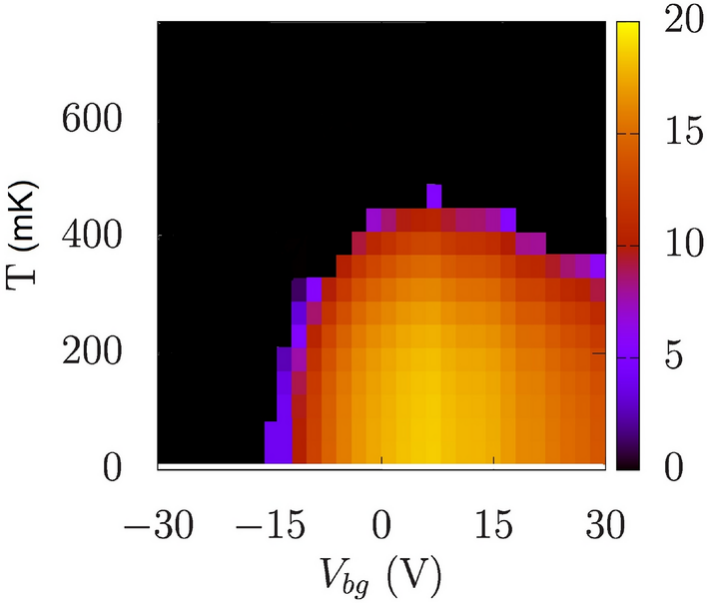
Superconducting energy gap $\Delta ^s_{xz/yz}$ as a function of back-gate voltage $V_{bg}$ and temperature *T*. The evaluated critical temperature at the maximum is 450 mK. Results for $m_l=0.05m_e$.

The characteristic dome-like shape of $\Delta _{l,n}(V_{bg})$, presented in Fig. 3, can be explained as resulting from the interplay between topology of the Fermi surface and symmetry of the order parameter. For this purpose we analyze the evolution of the Fermi surface with respect to the extended s-wave symmetry factor $\gamma _s(\textbf{k})$, presented in Fig. 4, when increasing the back gate voltage. For the electron density for which the system is non-superconducting only the $d_{xy}$ states are occupied [cf. Fig. 4a]. The superconducting state appears when the $d_{xz/yz}$ state crosses the Fermi level at particular $V_{bg}$. In the low electron concentration regime, the Fermi surface of the $d_{xz/yz}$ bands is located close to the $\Gamma$ point, in the center of the Brillouin zone [cf. Fig. 4b]. In this regime, the extended s-wave symmetry factor $\gamma _s(\textbf{k})=0.5(\cos (k_x)+\cos (k_y))$ [Eq. (15)], expressed in the wave vector space, is close to 1 and it does not influence the paring amplitude. In this regime the energy gap is enhanced with increasing electron concentration, due to the rise of density of states. However, as more and more electrons are added to the system, the Fermi surface expands and moves closer to the nodal lines of the extended s-wave symmetry, where the gap closes [cf. Fig. 4c,d]. The closer the Fermi surface is to the nodal lines the more the SC gap is going to be suppressed. At some point, the negative factor related to the gap symmetry starts to play a dominant role in relation to the positive factor related to the DOS enhancement. This leads to suppression of the $\Gamma ^s(\textbf{k})$ and weakening of the SC state. Consequently, the characteristic dome-like shape of $T_c(V_{bg})$ appears as presented in Fig. 5, where we display the temperature dependence of the extended s-wave component of the superconducting gap for the $d_{xz/yz}$ band. The evaluated critical temperature at the maximum is 450 mK, close to the experimentally measured value of approximately 300 mK^34^. Although in Fig. 5 we present the superconducting gap for the $d_{xz/yz}$ state, the determined critical temperature is the same for all the bands participating in the superconducting state, which is guaranteed by the Cooper pair hopping, determined by $J^{\prime }$

It is important to underline that in our model, the $d_{xy}$ bands do not play any significant role in superconducting phase. Moreover, the gap does not exhibit any nodal line as the decrease of $\Delta _{l,n}$ originates from the Fermi surface approaching the nodal lines of the extended s-wave $\gamma ^s(\textbf{k})$ factor but it never crosses them. Such nodal line crossing might results in the $s^{\pm }$ symmetry, but is not reached here. This is particularly important as measurements of tunneling and microwave conductivity in (100) LAO/STO are more in favor of a nodeless gap^52–54^.

### Effect of top-gating on superconductivity

We now focus on the superconducting gap when a top gate voltage is applied to the heterostructure. In the Schrödinger–Poisson approach, this specific electrostatic gating generates or reduces the electron concentration of the 2DEG, maintaining the charge neutrality of the system. In this case, the boundary condition on the right side is determined by the back gate voltage. In our calculations, we assume that the top gating may change the electron concentration by $\pm 0.5 \times 10^{13}$ $\hbox {cm}^{-2}$ from the reference value determined by the back gate.

**Fig. 6:**
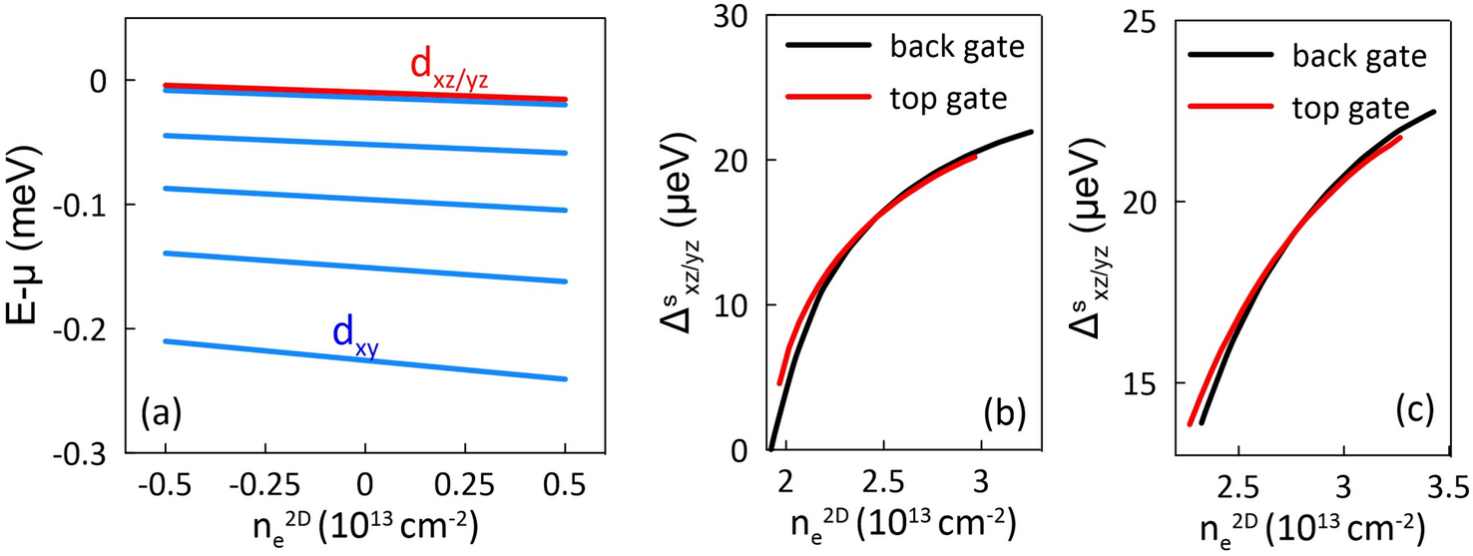
(**a**) Electronic structure as a function of doping with the top gate voltage. Panels (**b**, **c**) present the dominant extended *s*-wave component of superconducting gap for the $d_{xz/yz}$ bands calculated for the bottom (black) and top (red) gating, assuming in the latter case $V_{bg}=-4$ V (**b**) and $V_{bg}=0$ V (**c**).

Figure 6a demonstrates the electronic structure as a function of the change in the electron concentration induced by the top gating. Note that energies of both the $d_{xy}$ and $d_{xz/yz}$ bands depend linearly on the doping induced by the top gate. This linear behavior is observed regardless of the applied bottom gate voltage. Based on the determined electronic structure, for two cases, namely $V_{bg}=-4$ V and $V_{bg}=0$, we have evaluated the superconducting gaps as a function of doping. A comparison between both the top and bottom gate doping is presented in Fig. 6b,c, where we display only the dominant extended s-wave component of $\Delta _{xz/yz}^s$ as a function of the electron concentration $n_e^{2D}$.

Similarly to the experiment^34^, we observe that the value of $\Delta ^s_{d_{xz/yz}}$ at a specific $n_e^{2D}$ depends on the type of gating. Note, however, that the strength of this bifurcation is significantly lower than that observed experimentally, and moreover, for large $V_{bg}$, above the gap maximum, the bifurcation tends to disappear. We believe that this slight discrepancy is related to the simplified Schrödinger–Poisson approach applied in our model. In this approach, we do not consider the LAO layer of the heterostructure, treating it as a charged plane in order to fulfill the neutrality condition. In a real experiment, the voltage applied to the top gate, located close to the 2DEG, disturbs also the confinement potential by electrostatic means, which could lead to a more pronounced bifurcation as observed in the experiment. Note, moreover, that Ref.^34^ reports critical temperature changes, close to the $T_c$ maximum, exhibiting bistable behavior: increasing with doping via top gating and decreasing with back gating. In our case, the bifurcation significantly decreases close to the $T_c$ maximum, making this kind of observation impossible.

## Summary

We have considered the superconducting properties of the 2DEG at the LAO/STO interface within a microscopic model based on the Schrödinger–Poisson approach coupled with the real-space pairing model at the mean field level. Our calculations have been conducted with respect to the recent experiment with a double-gate field-effect device^34^.

In order to directly relate our results with the experimental data, first we have determined the electronic structure of the LAO/STO 2DEG under back gating, which then has been used to evaluate the superconducting gap. For the electron concentration regime reported in the experiment, we have reproduced the dome-like shape of the gap as a function of gate voltage (electron concentration) and explained it as resulting from the extended s-wave symmetry of the gap induced in the higher $d_{xz/yz}$ band. Our calculations with top gating have exhibited a bifurcation in the superconducting gap similar to that observed in the experiment, although its strength is significantly lower, which we have attributed to the simplified Schrödinger–Poisson approach used in our calculations.

Note that in our model we neglect SOC which is reported to be relatively strong at the LAO/STO interface^12–18^. The impact of SOC on the superconducting dome of 2DEG at the LAO/STO interface was analyzed in details in one of our previous papers^32^, within the three band approximation. It clearly demonstrates that in (001) LAO/STO interface, the SOC does not affect the appearance of the superconducting dome. Its shape changes slightly, but the dome still exists even for relatively strong SOC. For this reason, we argue that the effect of the SOC is negligible here and is not able to change the main finding of the paper that the superconducting dome results from the specific symmetry of the gap, which opens when the higher $d_{xz/yz}$ bands are occupied. As an additional argument, note that the electron density at which we observe the maximum of $\Delta_{l,n}$ in Fig. 3 corresponds relatively well with the electron density at which maxium of $T_c$ was reported in the experiment^34^.

In summary, our results strongly indicate that an unconventional symmetry of the gap in the form of extended s-wave type reproduces almost all features observed in the experiments and should be considered as a very probable theoretical scenario to explain the unconventional superconducting phase diagram of the 2DEG at the LAO/STO interface. Note that this hypothesis seems to be supported by recent measurements in LAO/STO-based Josephson junctions, which exhibit an unconventional Fraunhofer pattern with a local minimum at zero magnetic field^51,55,56^, characteristic for atypical gap symmetries.

Finally, we should underline that in our model the $d_{xy}$ states do not contribute to superconductivity. Thus none of the drawbacks occurring in the scenario based on the interband scattering, namely the fact that (i) the scattering occurs predominantly between the $d_{xz/yz}$ band and the higher-lying $d_{xy}$ state, while the lower-lying $d_{xy}$ states do not significantly affect superconductivity as well as (ii) the electronic structure that favors the specific alignment of electronic states with the Lifshitz transition related with the $d_{xy}$ band, occur in our model, shading light on the interpretation of recent experiments.

## Data Availability

The datasets used and/or analysed during the current study together with appropriate codes available from the corresponding author on request.
